# Austausch von Aniridie-IOL gegen individuelle Iris-IOL-Implantate

**DOI:** 10.1007/s00347-021-01447-9

**Published:** 2021-07-08

**Authors:** C. Mayer, D. Scharf, R. Khoramnia

**Affiliations:** Universitätsaugenklinik Heidelberg, Im Neuenheimer Feld 400, 69120 Heidelberg, Deutschland

**Keywords:** Okuläres Trauma, Irisprothese, Pupillenrekonstruktion, Irisdefekt, Ästhetik, Aphakie, Ocular trauma, Iris prosthesis, Pupil reconstruction, Iris defect, Esthetics, Aphakia

## Abstract

**Hintergrund:**

Es können 3 Gruppen an Irisprothesen zur chirurgischen Versorgung von Irisdefekten unterschieden werden: (1) segmentale Irisimplantate, (2) kombinierte Irisblenden-Intraokularlinsen (IOL) und (3) reine Irisimplantate. Die meisten Irisrekonstruktionen gehen zusätzlich mit einer Aphakiekorrektur durch sekundäre Linsenimplantation einher. Auch wenn primäre Ziele die Herstellung einer Pupille sowie die Besserung des Blendungsempfindens, der Kontrastsensitivität und der Sehschärfe sind, spielt das ästhetische Ergebnis eine nicht zu vernachlässigende Rolle.

**Ziel der Arbeit:**

Dargestellt werden funktionelle und ästhetische Ergebnisse nach Austausch von Aniridie-IOL-Implantaten gegen eine individuell angefertigte künstliche Iris in Kombination mit einer IOL.

**Material und Methoden:**

In dieser retrospektiven Studie mit 7 Augen von 7 Patienten wurde eine Irisblenden-IOL (Morcher GmbH, Stuttgart) aus medizinischen Gründen (Subluxation) gegen eine individuell hergestellte künstliche Iris aus Silikon (Artificial*Iris*, HumanOptics, Erlangen) in Kombination mit einer angenähten IOL ausgetauscht. Die Nachbeobachtungszeit betrug mindestens 3 Monate. Bestkorrigierter Fernvisus (BCVA), Endothelzellzahl (ECC), Komplikationen, Blendungsempfinden, das ästhetische Ergebnis und die Patientenzufriedenheit wurden evaluiert.

**Ergebnisse:**

BCVA und ECC zeigten keine statistisch signifikante Änderung zwischen prä- und postoperativ (*p* > 0,05). Es zeigte sich eine Dezentrierung des Iris-IOL-Implantats von 0,27 ± 0,19 mm 3 Monate postoperativ. Auf einer visuellen Analogskala (VAS) von 1 bis 10 (1 = gar nicht bis 10 = extrem zufrieden) wurde die Zufriedenheit mit dem Gesamtergebnis mit 8,6 ± 2,5 bewertet. Das subjektive Blendungsempfinden besserte sich auf 5,6 ± 3,5 und die subjektive ästhetische Beeinträchtigung auf 2,4 ± 2,0 auf der VAS (1 = gar nicht bis 10 = extrem stark). Die postoperativen Komplikationen umfassten eine vorübergehende intraokulare Hypotonie in zwei, einen Druckanstieg in zwei, eine Netzhautablösung und eine transiente Glaskörperblutung jeweils in einem Auge. Sechs von sieben Patienten würden den Eingriff wiederholen.

**Schlussfolgerung:**

Im Vergleich zu einem starren Aniridie-IOL-Implantat bietet der Austausch gegen eine individuell angefertigte künstliche Iris in Kombination mit einer IOL neben einem guten funktionellen gleichzeitig auch ein ästhetisch ansprechendes Ergebnis.

**Video online:**

Die Online-Version dieses Beitrags (10.1007/s00347-021-01447-9) enthält ein Video.

## Abstract (en)

**Background:**

Three groups of iris prostheses can be distinguished for the surgical treatment of iris defects: (1) segmental iris implants, (2) combined iris diaphragm intraocular lenses (IOL) and (3) pure iris implants. Most iris reconstructions are accompanied by aphakia correction with secondary IOL implantation. Although the primary goal is to create a pupil and to improve glare perception, contrast sensitivity and visual acuity, the esthetic result is also a relevant component.

**Objective:**

Functional and esthetic results after replacement of an aniridia IOL implant with a custom-made artificial iris with IOL.

**Material and methods:**

In this retrospective study with seven eyes from seven patients, an iris diaphragm IOL (Morcher GmbH, Stuttgart, Germany) was exchanged for medical reasons (subluxation) against a custom-made artificial iris made of silicone (Artificial*Iris*, HumanOptics, Erlangen, Germany) in combination with a sutured IOL. The follow-up period was at least 3 months. Best corrected distance visual acuity (BCVA), endothelial cell count (ECC), complications, glare perception as well as esthetic outcome and patient satisfaction were evaluated.

**Results:**

The BCVA and ECC showed no statistically significant change between the preoperative and postoperative values (*p* > 0.05). There was a decentration of the iris IOL implant of 0.27 ± 0.19 mm three months postoperatively. On a visual analogue scale (VAS) from 1 to 10 (1 = not satisfied at all, 10 = extremely satisfied), satisfaction with the overall result was rated 8.6 ± 2.5. Subjective glare perception improved to 5.6 ± 3.5 and subjective esthetic impairment improved to 2.4 ± 2.0 on the VAS (1 = none, 10 = extremely strong). Postoperative complications included transient intraocular hypotonia in two eyes, intraocular pressure increase in two eyes, retinal detachment and transient vitreous hemorrhage in one eye each. Of the seven patients six would repeat the procedure.

**Conclusion:**

Compared to a rigid aniridia IOL implant, replacement with a custom-made artificial iris in combination with an IOL provides not only a good functional result but simultaneously also an esthetically pleasing result.

## Hintergrund und Fragestellung

Patienten mit erworbenen Irisdefekten und Aphakie haben oft einen hohen subjektiven Leidensdruck. Dieser resultiert u. a. aus der reduzierten unkorrigierten Sehschärfe, einem ausgeprägten Blendungsempfinden, der Abnahme der Kontrastsensitivität und nicht zuletzt aus der ästhetischen Beeinträchtigung [7, 28]. Eine intakte Regenbogenhaut trägt zur maximal erreichbaren Sehqualität bei, reduziert das einfallende Licht und verbessert die Tiefenschärfe [17, 23]. Ursache für die erworbenen Defekte sind meistens Folgeschäden nach stumpfen oder perforierenden Traumata. Durch einen Iriskontaktlinsen-Trageversuch kann präoperativ die Blendungsreduktion simuliert werden [16, 27]. Die zugrunde liegenden Komorbiditäten nach einem Trauma beeinflussen die postoperative Sehschärfe [22, 23].

**Figure 1:**
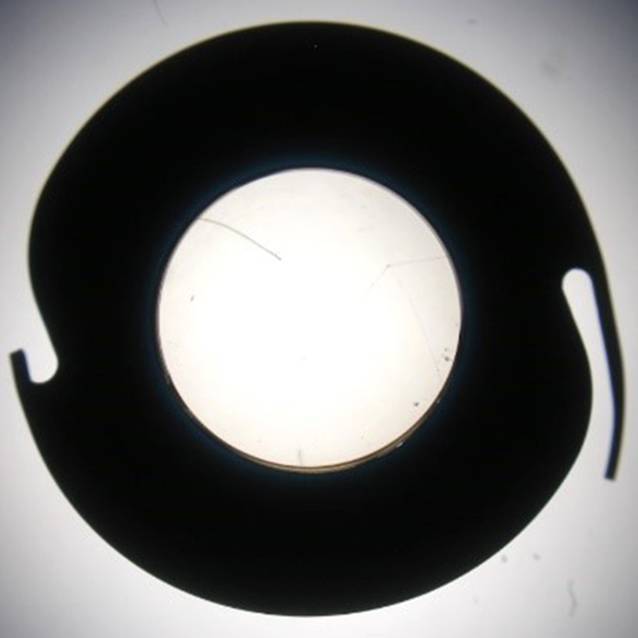

**Figure 2:**
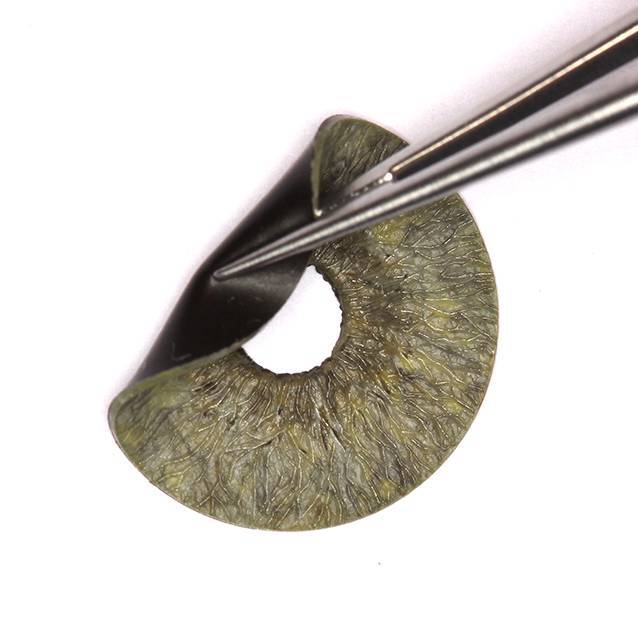

Die erste Implantation einer Irisprothese in die Vorderkammer erfolgte 1964 von Peter Choyce [3]. Heutzutage lassen sich 3 Gruppen unterscheiden, die alle in die Hinterkammer implantiert werden:

1. die Gruppe der segmentalen Irisimplantate (Kapselspannringe mit Blendensegmenten und Teilirisprothesen).
2. Die Gruppe der Irisblenden-IOL (Morcher GmbH, Stuttgart, Deutschland) sind auf die 1991 erstmals angebotene erste Irisblenden-IOL von R. Sundmacher zurückzuführen [24, 25]. Es handelt sich hierbei um eine IOL mit einem Irisdiaphragma aus schwarzem Polymethylmethacrylat (PMMA, Aniridieimplantat Type 68) von 10 mm Durchmesser mit einer gebogenen Haptik und einer zentralen Öffnung mit oder ohne brechender Optik ([23]; Abb. 1). Weiter fällt in diese Gruppe auch die Irisrekonstruktionslinse Modell C1/F1 bikonvex (Ophtec BV, Groningen, Niederlande). Sie bietet eine gefächerte Farbvielfalt (> 1200 Farbkombinationen), um dem verbliebenen Irisgewebe oder der Iris des Partnerauges zu entsprechen. Alternativ existiert noch eine Irisprothese Modell C0/F0 ohne optische Korrektur.
3. Ästhetisch vielversprechende Ergebnisse, die dem anatomischen Normalbefund nah kommen, liefert die dritte Gruppe [6]: Bei der Artificial*Iris* (HumanOptics, Erlangen, Deutschland) handelt sich um ein flexibles, individuell angefertigtes Irisdiaphragma aus Silikon ohne zentrale Optik (Abb. 2; [27]). Dieses Implantat kann im Rahmen einer Kataraktoperation kombiniert mit einer IOL oder aber in bereits pseudophaken Augen eingesetzt werden. Die Implantation erfolgt in den Kapselsack oder über eine Nahtfixation an Sklera oder Iris [27].

Für den Ersatz des ursprünglichen Aniridielinsenimplantats fiel die Entscheidung auf das Artificial*Iris*-Modell von HumanOptics, da dieses eine individuelle IOL-Auswahl in Kombination mit der Irisprothese zulässt und gleichzeitig gute ästhetische Ergebnisse verspricht. Dies wurde in der hier vorliegenden Arbeit untersucht.

## Material und Methoden

**Figure 3:**
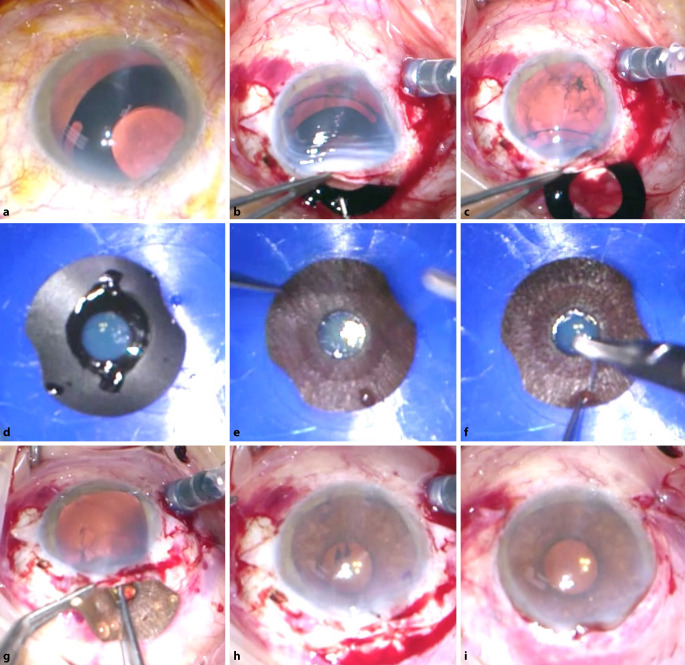

Diese retrospektive Studie umfasst 7 Patienten, die sich im Zeitraum von 2014 bis 2020 an den Universitätsklinika TU München und Heidelberg mit Problemen einer Irisprothese vorstellten. Der Iris- und Linsendefekt war in allen Fällen zuvor mit einer schwarzen Irisblenden-IOL (Morcher, Implantat Typ 68) versorgt worden (Abb. 2). Alle Patienten wurden umfassend ophthalmologisch untersucht inklusive Erhebung des bestkorrigierten Fernvisus (BCVA), des Augeninnendruckes und des Befundes von vorderem und hinterem Augenabschnitt. Es erfolgte eine Fotodokumentation. Die Berechnung der IOL erfolgte mit dem IOL Master 700 (Carl Zeiss Meditec AG, Jena, Deutschland). Ein Korrekturfaktor wurde nicht angewendet, da er nicht erforderlich ist [5]. Die Endothelzellzahl (ECC) wurde mit dem Endothelmikroskop EM-3000 (Tomey, Nagoya, Japan) gemessen. Da die Daten für den BCVA und die ECC nach Inspektion der Histogramme nicht normalverteilt waren, wurden Unterschiede zwischen prä- und postoperativ mit dem nichtparametrischen Wilcoxon-Test untersucht. Die Patienten beantworteten prä- und postoperativ einen Fragebogen zum subjektiven Blendungsempfinden, zur ästhetischen Beeinträchtigung, zur Zufriedenheit mit dem Gesamtergebnis und zur Änderung der Lebensqualität. Da es zu diesen Fragestellungen keinen validierten Fragebogen gibt [28], wurden die Antworten auf einer visuellen Analogskala von 1 bis 10 (1 = gar nicht bis 10 = extrem stark bzw. extrem zufrieden) angegeben. Die stets medizinische Indikation zur Entfernung bzw. zum Austausch des Implantats kann in Tab. 1 eingesehen werden. Über die Gefahr einer potenziellen Abnahme des BCVA postoperativ wurde im Rahmen der ausführlichen Darlegung der Operationsrisiken mit aufgeklärt [6]. Die Untersuchung wurde im Einklang mit nationalem Recht sowie in Übereinstimmung mit der Deklaration von Helsinki von 1975 (in ihrer aktuellen, überarbeiteten Fassung) durchgeführt. Bisher wurde die Artificial*Iris* durch den Operateur (CM), der alle Patienten in der vorliegenden Studie operierte, bei mehr als 120 Patienten mit unterschiedlichen Operationstechniken implantiert [6]. Die einzeitige, operative Vorgehensweise mit Explantation des Aniridie-IOL-Implantats und Implantation der neuen Prothese ist stellvertretend in Video 1 und Abb. 3 dargestellt: Hierbei wurde in allen Fällen die künstliche Iris mit der IOL an der Rückfläche der Irisprothese extraokular vernäht und dann als „Sandwich“ implantiert [8]. In einer Laborstudie untersuchten wir die Auswirkung einer Nahtfixierung der IOL an die Artificial*Iris*. Es zeigten sich dabei keine optischen Beeinträchtigungen [9].

**Table 1:** 

*n*	m/w	Alter	Auge	Indikation zur Irisblenden-IOL	Zeit zwischen Implantation und Austausch der Irisblenden-IOL (Jahre)	Indikation zum Austausch der Irisblenden-IOL	Operationsdauer (min)	Vor Implantation der künstlichen Iris-IOL					≥ 3 Monate nach Implantation der künstlichen Iris-IOL						Postoperative Besonderheiten
								Subjektive Blendung	Subjektive ästhetische Beeinträchtigung	BCVA (logMAR)	IOD (mm Hg)	ECC (Zellen/mm^2^)	Subjektive Blendung	Subjektive ästhetische Beeinträchtigung	BCVA (logMAR)	IOD (mm Hg)	ECC (Zellen/mm^2^)	Pupillenzentrierung (mm)	
								1 gar nicht bis 10 extrem stark					1 gar nicht bis 10 extrem stark						
1	m	13	RA	Z. n. Contusio bulbi mit einem Feuerwerkskörper	1	Psychische Belastung, ästhetisch stark beeinträchtigt, „kam bei Frauen nicht gut an“	60	3	5	1,3	10	2215	8	1	1,5	14	2128	0,16	–
2	w	45	RA	Z. n. Iridektomie und Phakoemulsifikation mit Hinterkammerlinse bei kongenitaler Katarakt	36	Subluxation nach nasal inferior, subjektiv starke Blendung, hoher Leidensdruck, Iriskontaktlinsen als Notlösung und schlechte Verträglichkeit	60	8	10	< 1,3	18	1686	3	6	< 1,3	18	2016	0,36	Leichte Glaskörperblutung, Pigmentdispersion, Hyposphagma, drucksenkende Tropfen bei IOD von 20–30 mm Hg, bereits präoperativ Glaukomverdacht
3	m	31	LA	Z. n. Keratoplastik, Subtotale Aniridie bei Z. n. perforierender Verletzung, V. a. Amblyopie	28	Subluxation nach temporal inferior, Leidensdruck bei Visusverschlechterung	75	8	1	1,7	14	1873	3	1	1,4	16	1928	0,63	Zunächst Hypotonie, langsamer Druckaufbau bis Hypertonie auf 59 mm Hg, Glaukomtherapie
4	m	79	RA	Z. n. perforierender Verletzung	14	Subluxation nach temporal superior	55	5	5	1,3	14	2451	5	3	0,5	12	2451	0,36	–
5	m	50	LA	Z. n. Phakoemulsifikation mit Hinterkammerlinsen-Implantation 2016 bei Z. n. perforierender Verletzung 1987 (Arbeitsunfall)	Nicht bekannt	Subluxation nach nasal, subjektiv starke Blendung	22	7	3	0,1	14	1698	1	1	0,1	20	928	0,09	–
6	m	29	LA	Z. n. perforierender Verletzung	Nicht bekannt	Subluxation, Irisblenden-IOL hängt nur noch locker an einer Skleraseite, Notfalloperation	75	8	1	HBW	18	2110	9	4	0,2	29	1969	0,18	Zunächst Hypotonie, Druckaufbau, drucksenkende Tropfen bei IOD von 29 mm Hg, TEM bei Sicca
7	w	41	RA	Traumatische subtotale Aniridie bei Z. n. Keratoplastik 1993 und Z. n. Amotiooperation 1989 bei Z. n. perforierender Verletzung in der Kindheit mit Eiszapfen	30	Subluxation nach inferior, subjektiv starke Blendung	100	7	8	1,4	10	Nicht möglich	10	1	Fingerzählen bei Amotio	10	Nicht möglich	0,12	IOL-AI-Austausch bei Hornhautdekompensation mit Re-KPL, Glaukomtherapie, 2 Monate postoperativ ppV + Silikonöl bei Amotio, 4 Monate postoperativ Re-ppV + Silikonöl bei Re-Amotio

*BCVA* bestkorrigierter Fernvisus, *ECC* Endothelzellzahl, *HBW* Handbewegung, *IOD* Augeninnendruck, *IOL* Intraokularlinse, *KPL* Keratoplastik, *LA* linkes Auge, *ppV* Pars-plana-Vitrektomie, *RA* rechtes Auge

## Ergebnisse

Der Irisdefekt war bei 5 Augen durch eine perforierende Verletzung, bei 1 Auge durch eine Contusio bulbi und bei 1 Auge durch mehrfache Operationen bei kongenitaler Katarakt hervorgerufen worden (Tab. 1). In 6 Augen (86 %) war die Irisblenden-IOL bei Erstvorstellung subluxiert.

**Figure 4:**
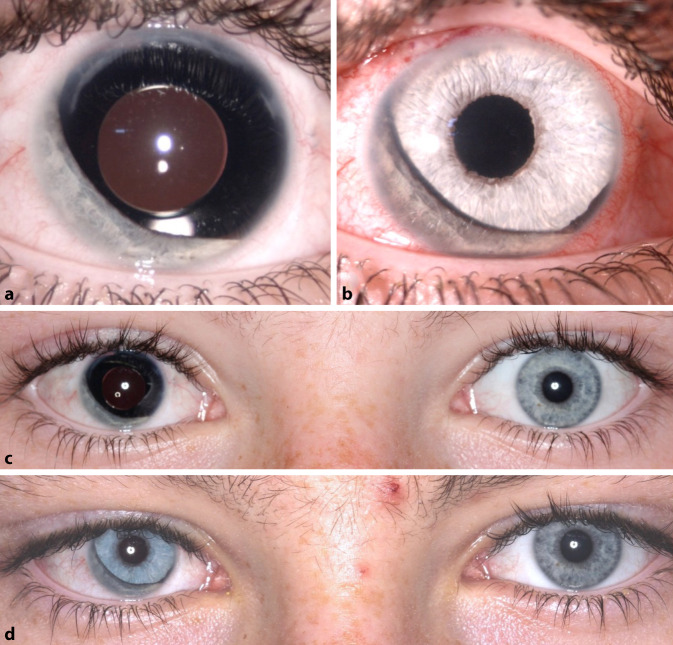

Das mittlere Alter der hier untersuchten Patienten lag bei 41,1 ± 20,7 Jahren. Die Nachbeobachtungszeit lag im Mittel bei 5 ± 3,3 Monaten (Minimum 3 Monate, Maximum 12 Monate). Die Ergebnisse hinsichtlich des BCVA, des Augeninnendrucks, der ECC und der Patientenzufriedenheit sowie Symptome anhand eines Fragebogens werden in Tab. 1 zusammengefasst. Hinsichtlich des BCVA und der ECC zeigten sich keine statistisch signifikanten Änderungen beim Vergleich zwischen prä- und postoperativ (*p* > 0,05). Intraoperativ stellte sich das kombinierte Iris-IOL-Implantat immer gut zentriert dar (Abb. 4 und 5). Die Pupillenzentrierung wurde in der Nahaufnahme 3 Monate postoperativ mit dem Heidelberg Eye Explorer (Heyex, Heidelberg Engineering, Heidelberg) gemessen. Der Abstand zwischen dem geometrischen Zentrum der Hornhaut und dem Zentrum der neu geschaffenen Pupille wurde in Pixeln gemessen und in Millimeter umgerechnet [28]. Es zeigte sich eine Dezentrierung von 0,27 ± 0,19 mm drei Monate postoperativ (Tab. 1).

**Figure 5:**
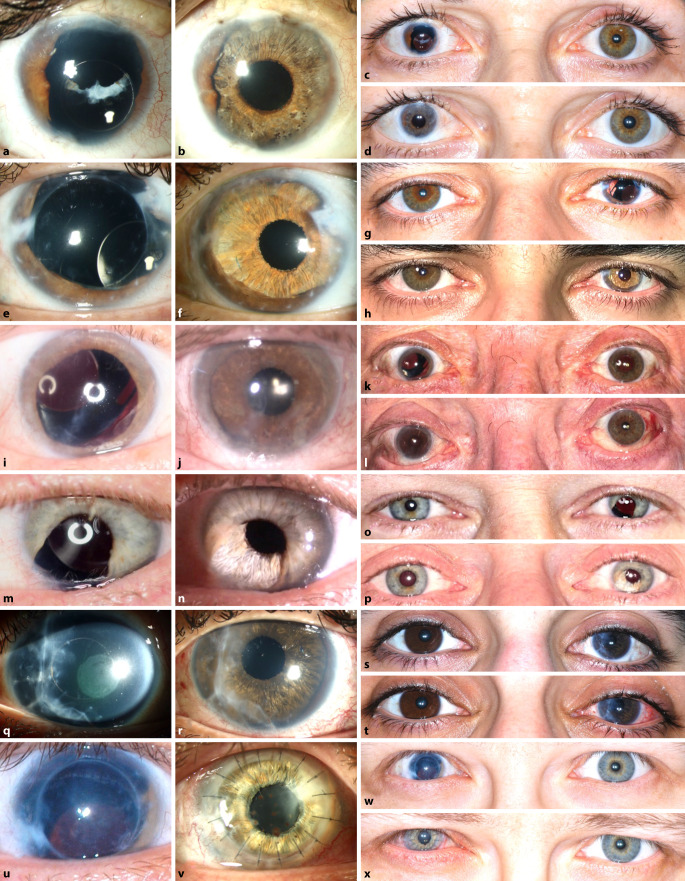

Präoperativ bewerteten die Patienten das subjektive Blendungsempfinden mit 6,6 ± 1,9 Punkten auf der visuellen Analogskala. Postoperativ besserte sich das Blendungsempfinden auf 5,6 ± 3,5 Punkte. Die ästhetische Beeinträchtigung wurde vor dem Austausch mit 4,7 ± 3,4 und postoperativ mit 2,4 ± 2,0 Punkten bewertet. Die Gesamtzufriedenheit der ausgewerteten Patienten mit dem Ergebnis lag bei 8,6 ± 2,5 Punkten. Sechs von 7 Patienten würden den Austausch nochmals durchführen lassen, wenn sie neu entscheiden könnten. Angesichts des komplexen postoperativen Verlaufes mit mehreren Re-Operationen würde ein Patient die Operation nicht nochmals durchführen. Im Mittel lag die Verbesserung der Lebensqualität bei 7,4 ± 1,7 Punkten (*n* = 5/7). In Abb. 4 ist beispielhaft das ästhetische Ergebnis bei fast vollständigem Irisdefekt dargestellt.

## Diskussion

Das primäre Ziel des hier beschriebenen operativen Eingriffs sollte die Behandlung des (sub)luxierten Iris-IOL-Implantates sein. Ein gut situiertes, stabiles, älteres Iris-IOL-Implantat sollte ohne Beschwerden, hohen Leidendruck oder triftigen Grund nicht alleine wegen des ästhetischen Aspekts ausgetauscht werden. Alle eingeschlossenen 7 Patienten, bei denen Probleme zu einem Wechsel der Prothesen geführt haben, waren Jahre zuvor mit einer schwarzen Irisblenden-IOL versorgt worden. Einerseits kann die initiale Verwendung dieses Modells dadurch begründet sein, dass der Operateur mit diesen Irisprothesen vertraut ist und auf diese zugreifen konnte, sowie andererseits dadurch, dass die Artificial*Iris* zum Zeitpunkt der Erstversorgung noch nicht zur Verfügung stand. Die Morcher GmbH entwickelte verschiedene Prothesen unterschiedlicher Größe und Form. Morcher Irisblenden-IOL sind steife Implantate, die durch eine große sklerokorneale Inzision von 150–180° eingesetzt werden [23, 27]. Vorteilhaft ist, dass es keiner separaten IOL bedarf [23]. Die Implantation der Irisblenden-IOL Morcher Typ 67B liefert bezüglich Visus und Blendungsempfinden gute funktionelle Ergebnisse [18, 20]. Eine Refixierung der subluxierten Irisblenden-IOLs wäre ebenfalls denkbar gewesen, ist aber operationstechnisch auch nicht weniger komplex. Unter Berücksichtigung verschiedener Faktoren (Gefahr eines Haptikdefektes wegen des steifen Materials, hinreichende Erfahrung mit der neuen Prothese und Möglichkeit der Anwendung, bekannte bessere ästhetische Ergebnisse, zur Verfügung stehende standardisierte Operationstechnik) wurde ein Austausch bevorzugt [8, 27, 28]. Sowohl die funktionellen als auch kosmetischen Ergebnisse fallen bei Verwendung der Artificial*Iris* sehr gut aus [4, 6, 11, 22]. Die Faltbarkeit von Silikon erlaubt prinzipell die Implantation durch eine kleinere Inzision [1, 23]. Ein weiterer Vorteil ist, dass die Artificial*Iris* maßangefertigt wird. Das ästhetische Ergebnis mit einer schwarzen Prothese ist für die Patienten nicht ideal [20]. Die farbliche Orientierung am Partnerauge führt zu einem besseren ästhetischen Ergebnis, nicht zuletzt durch das in die Tiefe individuell eingearbeitete Farbpigment [11, 21, 26].

Postoperativ kann es zu einer Dezentrierung bzw. Subluxation der Irisprothese, zu einem transienten Anstieg des Augeninnendrucks oder einer rezidivierenden Blutung aus dem Ziliarkörper kommen [6]. Als häufigste postoperative Komplikation ist ein erhöhter Augeninnendruck zu nennen [26]. Das Vorliegen eines Glaukoms oder eines Endothelzellschadens sind präoperativ zu berücksichtigen [22, 26]. Hierbei kann das Implantat den Ziliarkörper mechanisch irritieren und durch eine Entzündung oder eine Kammerwasserabflussstörung einen Druckanstieg herbeiführen [19]. Ein früher transienter Augeninnendruckanstieg lässt sich adäquat mit drucksenkender Medikation behandeln [12]. Als weitere postoperative Komplikationen werden die Glaskörperblutung, die Netzhaut- oder Aderhautablösung, die Hypotonie, die Endophthalmitis, der chronische Reizzustand und das zystoide Makulaödem genannt [12, 23]. Langzeitkomplikationen lassen sich nur schwierig einordnen, da der bereits vorgelegene ursächliche Schaden nicht immer sicher vom induzierten Schaden differenziert werden kann [26]. In Langzeitbeobachtungen wurde ein Restirisretraktionssyndrom („residual iris retraction syndrome“ [RITS]) beschrieben [2, 13]. Bei verbliebener Restiris kann sich eine Nachblutung oder ein Nachdunkeln der natürlichen Iris auf das ästhetische Ergebnis auswirken [10]. Wegen dieser möglichen mechanischen Irritationen und einer potenziellen Pigmentdispersion gibt es Überlegungen, auch die unnötige Restiris zu entfernen. Resultierende Komplikationen können im Zusammenhang mit der Irisprothese selbst auftreten oder auch Folge der kongenitalen oder traumatischen Aniridie sein [23]. Die Komplikationsrate sinkt mit zunehmender Erfahrung und der Lernkurve des Operateurs [12, 15]. Es ist prinzipiell zu empfehlen, die Irisrekonstruktion erst nach Ausheilung anderer Pathologien vorzunehmen. Im Akutfall sind direkt nach einem Trauma in der Regel keine Implantate sofort greifbar. Um in diesem Fall dennoch ein vorübergehendes Irislinsendiaphragma zu erhalten – um z. B. eine Silikonölfüllung ohne Endothelkontakt zu erreichen –, sind provisorische oder dauerhafte Irisgitternähte möglich [14].

Die postoperative Nachsorge des Patienten ist wichtig und umfasst regelmäßige Kontrollen des Augeninnendrucks, des richtigen Sitzes des Implantates, das Achten auf eine Pigmentdispersion und eine Überwachung der ECC. Die Abnahme der ECC ist vergleichbar mit der Abnahme der ECC nach einer üblichen Kataraktoperation [12]. Die Verringerung des Blendungsempfindens und die Verbesserung der Kontrastsensitivität werden durch die Reduktion der Pupillenöffnung erreicht [2, 10, 11]. Die Zentrierung der Pupille ist postoperativ ein ästhetisch wichtiger Aspekt [28]. Bei einem Patienten dieser Serie ist die Pupille leicht nach inferior versetzt. Dies ist am ehesten auf intraokulares Narbengewebe im Bereich des Sulcus ciliaris zurückzuführen, welches sich nach mehreren Operationen aufgrund einer kongenitalen Katarakt entwickelt hatte.

Die Weiterentwicklung der Irisimplantate von rigiden zu flexiblen Prothesen ist als ein Meilenstein zu betrachten. Die Entscheidung zu einer maßangefertigten künstlichen Iris erfordert nicht zuletzt durch die präzise Planung einen Mehraufwand in der Vorbereitung und Patientenbetreuung. Der Eingriff sollte nie allein aus ästhetischen Gründen indiziert werden. Die vorliegende Arbeit belegt, dass der Austausch einer starren Irisprothese gegen die individuelle flexible künstliche Iris in Kombination mit einer IOL visuell keine Nachteile mit sich bringt und gleichzeitig eine hohe Patientenzufriedenheit sowie gute ästhetische Ergebnisse bietet.

## Fazit für die Praxis

- Ein gut situiertes, stabiles, älteres Iris-IOL-Implantat sollte ohne Beschwerden oder triftigen Grund nicht alleine wegen des ästhetischen Aspekts gegen die vorgestellte, individuell angefertigte Prothese ausgetauscht werden.
- Die meist komplexe Ausgangssituation erfordert eine sehr detaillierte Aufklärung des Patienten mit einer sorgfältigen Planung der jeweiligen Operationstechnik zur Irisrekonstruktion.
- Auch im Falle von Problemen mit einem anderen Irisimplantat stellt die Artificial*Iris* eine funktionell gute und gleichzeitig ästhetisch ansprechende Behandlungsmethode von Irisdefekten dar.

## Supplementary Information




